# Alterations in the Gut Microbiome and Metabolisms in Pregnancies with Fetal Growth Restriction

**DOI:** 10.1128/spectrum.00076-23

**Published:** 2023-05-18

**Authors:** Zixin Tao, Yun Chen, Fang He, Jiawei Tang, Limei Zhan, Haoyue Hu, Ziling Ding, Shenghang Ruan, Yutao Chen, Beier Chen, Yan Wang, Xiaoling Guo, Liwei Xie, Mei Zhong, Qitao Huang

**Affiliations:** a Department of Obstetrics and Gynecology, Nanfang Hospital, Southern Medical University, Guangzhou, Guangdong, China; b Department of Obstetrics and Gynecology, The Third Affiliated Hospital of Guangzhou Medical University, Guangzhou, Guangdong, China; c The Second School of Clinical Medicine, Southern Medical University, Guangzhou, Guangdong, China; d Foshan Women and Children Hospital Affiliated to Southern Medical University, Foshan, Guangdong, China; e The First School of Clinical Medicine, Southern Medical University, Guangzhou, Guangdong, China; f State Key Laboratory of Applied Microbiology Southern China, Guangdong Provincial Key Laboratory of Microbial Culture Collection and Application, Guangdong Open Laboratory of Applied Microbiology, Institute of Microbiology, Guangdong Academy of Sciences, Guangzhou, Guangdong, China; g Department of Obstetrics and Gynecology, The First People’s Hospital of Foshan, Foshan, Guangdong, China; Wayne State University

**Keywords:** fetal growth restriction, gut microbiota, metabolism, multi-omics, placenta

## Abstract

Fetuses diagnosed with fetal growth restriction (FGR) are at an elevated risk of stillbirth and adulthood morbidity. Gut dysbiosis has emerged as one of the impacts of placental insufficiency, which is the main cause of FGR. This study aimed to characterize the relationships among the intestinal microbiome, metabolites, and FGR. Characterization was conducted on the gut microbiome, fecal metabolome, and human phenotypes in a cohort of 35 patients with FGR and 35 normal pregnancies (NP). The serum metabolome was analyzed in 19 patients with FGR and 31 normal pregnant women. Multidimensional data was integrated to reveal the links between data sets. A fecal microbiota transplantation mouse model was used to determine the effects of the intestinal microbiome on fetal growth and placental phenotypes. The diversity and composition of the gut microbiota were altered in patients with FGR. A group of microbial species altered in FGR closely correlated with fetal measurements and maternal clinical variables. Fecal and serum metabolism profiles were distinct in FGR patients compared to those in the NP group. Altered metabolites were identified and associated with clinical phenotypes. Integrated multi-omics analysis revealed the interactions among gut microbiota, metabolites, and clinical measurements. Microbiota from FGR gravida transplanted to mice progestationally induced FGR and placental dysfunction, including impaired spiral artery remodeling and insufficient trophoblast cell invasion. Taken together, the integration of microbiome and metabolite profiles from the human cohort indicates that patients with FGR endure gut dysbiosis and metabolic disorders, which contribute to disease pathogenesis.

**IMPORTANCE** Downstream of the primary cause of fetal growth restriction are placental insufficiency and fetal malnutrition. Gut microbiota and metabolites appear to play an important role in the progression of gestation, while dysbiosis induces maternal and fetal complications. Our study elaborates the significant differences in microbiota profiles and metabolome characteristics between women with FGR and normal pregnancies. This is the first attempt so far that reveals the mechanistic links in multi-omics in FGR, providing a novel insight into host-microbe interaction in placenta-derived diseases.

## INTRODUCTION

Fetal growth restriction (FGR) is a pathological condition in which a fetus fails to achieve its expected growth potential. The most popular definition of FGR is an estimated fetal weight or abdominal circumference that is less than the 10th percentile for gestation age (1). Perinatal complications have been identified as associated with FGR, including stillbirth, cerebral injuries, metabolic dysfunctions, elevated risk of cardiovascular diseases, and obesity in adulthood (2–5). It is estimated that in low- and middle-income countries, the prevalence of FGR has reached 19.3%, which places an enormous burden on the already overburdened health care system (6). The current understanding of the etiology of FGR involves maternal medical conditions, placental disorders, and genetic diseases. Although the primary pathophysiologic mechanisms are different, suboptimal uterine-placental perfusion and fetal malnutrition are considered the shared stage of pathogenesis (1, 7, 8).

Recent studies have linked gut microbiota (GM) to the placenta. GM acts as an information hub at the host-environment interface that integrates environmental and host-derived signals to maintain the balance of metabolic and immune systems (9–11). A GM disorder activates inflammatory pathways and induces placental impairments (12). Passing through the leaky gut, bacteria may either translocate themselves to the placenta or modulate its structure and function via specific metabolites (13, 14). Bacterial signals were detected in placentas from both preeclamptic women and the preeclamptic mouse model induced by fecal microbiota transplantation (FMT) (15). Gut microbiota-derived metabolites, such as short-chain fatty acids (SCFAs) and bile acids, circulate in the intestine, serum, and several extraintestinal organs, modulating various physiological processes (16, 17). Tu et al. first characterized the altered maternal gut microbiota in FGR (18). However, this correlation was derived from observational study and single-omics. The underlying mechanisms and microbial-induced host response remain largely unknown.

This study aimed to elucidate the link between gut microbiota and FGR using 16S rRNA sequencing and metabolomic analyses. Moreover, an FMT mouse model was used to validate the effect of gut dysbiosis.

## RESULTS

### Participant information.

The 16S rRNA sequences and metabolomes of fecal samples were obtained from 70 individuals, comprising 35 patients with FGR (FGR group) and 35 normal pregnancies (NP group). The NP group were characterized by uncomplicated pregnancies with normal-term deliveries, with estimated fetal weight (EFW) between the 10th to 90th percentile and birth weight ranging from 2,500 g to 4,000 g. The clinical characteristics of all of the pregnant women were shown in Table 1. Serum metabolomes were analyzed in 31 NP subjects, and 19 patients with FGR included in the above groups; the clinical features of the cohort are shown in Table S1 in the supplemental material. The two groups were similar in age, body mass index (BMI), and gravidity (Table 1). Levels of systolic and diastolic blood pressures, alanine aminotransferase (ALT), and aspartate aminotransferase (AST) were higher in the FGR group than in the NP group, whereas albumin (ALB) and total bilirubin (TBiL) displayed the opposite. Notably, these characteristics, although different, were within normal ranges for both subjects with or without FGR. In addition, pregnancies in the NP group had a median parity of one, and 14 (40%) were nulliparous, while the FGR group had a median parity of zero, among which 25 were nulliparous (71.4%). However, parity is a high-risk factor for low birth weight and small size for gestational age, which cannot be balanced in this study (19). The serum albumin level is decreased in the FGR group, indicating an inferior nutritional state. The biparietal diameter (BPD), head circumference (HC), abdominal circumference (AC), femur length (FL), and amniotic fluid volume (AFV) were measured with ultrasound. As expected, neonatal weight (NW) and the above measurements significantly decreased in the FGR group.

**TABLE 1:** Clinical characteristics of the study cohort^*a*^

Feature^*b*^	NP group (*n* = 35)	FGR group (*n* = 35)	*P* value
Maternal feature			
Age (yr)	29.0 (26.0−33.0)	29.2 (27.0−32.0)	0.653
Gestational age (w)	39.5 (39.0−41.0)	38.0 (36.5−38.5)	<0.001
Wt (kg)	66.2 (62.1−72.1)	64.5 (55.9−68.6)	0.0826
Ht (cm)	158 (155−160)	156 (153−158)	0.0797
BMI (kg/m^2^)	27.03 ± 3.13	25.97 ± 3.74	0.203
G	2 (1−3)	2 (1−2)	0.1077
P	1 (0−1)	0 (0−1)	0.0121
SBP (mmHg)	117 (112−126)	127 (121−138)	0.001
DBP (mmHg)	71 (64−75)	75 (70−85)	0.0017
PLT (×10^9^/L)	231.0 ± 58.0	243.5 ± 59.6	0.3797
ALT (U/L)	10.1 (8−12)	12.8 (9.0−18.2)	0.0041
AST (U/L)	14.5 (12.7−17.4)	19 (14.1−22.3)	<0.001
ALP (U/L)	156 (123−173)	136 (111−179)	0.2289
ALB (g/L)	36.29 ± 2.043	34.85 ± 3.750	0.0496
TBA (μmol/L)	3.4 (2.6−6.5)	3.1 (2.4−5.8)	0.7326
TBil (μmol/L)	8.0 (7.0−9.7)	6.9 (5.5−8.1)	0.0038
DBil (μmol/L)	1.4 (1.1−1.9)	1.3 (1.1−1.9)	0.4233
Cr (μmol/L)	45.0 (43.0−49.0)	51.0 (42.0−59.0)	0.0509
Fetal feature			
Fetal gender			0.6145
Male	22	24	
Female	13	11	
NW (g)	3,270 (3,020−3,590)	2,120 (1,950−2,420)	<0.0001
BPD (mm)	92.0 (86.0−95.0)	81.0 (75.0−86.0)	<0.0001
HC (mm)	329 (317−335)	296 (274−307)	<0.0001
AC (mm)	337 (325−345)	280 (257−292)	<0.0001
FL (mm)	72 (69−73)	62 (58−66)	<0.0001
AFV (mm)	48 (42−55)	38 (30−45)	0.0028

a Data were assessed for normality using the Shapiro-Wilk normality test and presented as mean ± SD or median (interquartile range). For normally distributed data, Student's *t* test was performed between two groups. For nonnormal distributed data, Mann-Whitney U test was performed. For the comparison of fetal gender, chi-square test was conducted.

b BMI, body mass index; G, gravidity; P, parity; SBP, systolic blood pressure; DBP, diastolic blood pressure; PLT, platelet; ALT, alanine aminotransferase; AST, aspartate aminotransferase; ALP, alkaline phosphatase; ALB, albumin; TBil, total bilirubin; DBil, direct bilirubin; Cr, creatinine; NW, neonatal weight; BPD, biparietal diameter; HC, head circumference; AC, abdominal circumference; FL, femur length; AFV, amniotic fluid volume.

### Diversity and composition of maternal gut microbiota.

**FIG 1:**
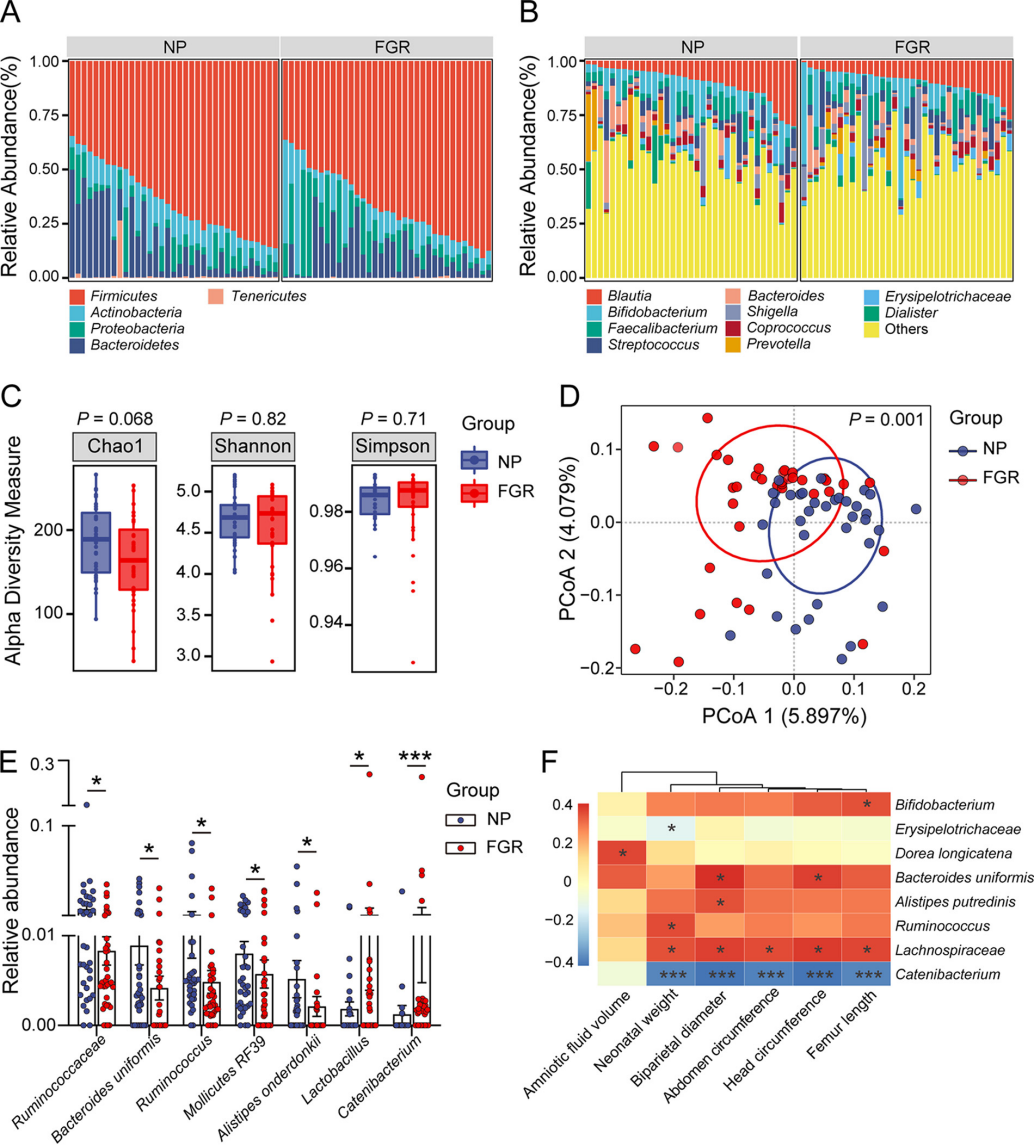
Gut microbiota profile of patients with FGR and normal pregnant women. (A) Component proportion of bacteria at phyla level in the two groups. (B) The composition of bacteria at species level. (C) α-Diversity of the two group. (D) β-Diversity based on unweighted UniFrac distances. (E) The relative abundances of significantly differentially abundant gut bacteria between the two groups. The *P* value was adjusted with FDR. (F) Correlation heatmap between fetal measurements and the abundant bacterial species. Data were presented as median and interquartile range (IQR) in panels C and E, and Wilcoxon rank-sum test was used for statistical analysis. Spearman’s correlation test was used for statistical analysis. *, *P *< 0.05; **, *P *< 0.01; ***, *P *< 0.001. NP, *n* = 35; FGR, *n* = 35.

To characterize the gut microbiota profiles, we conducted 16S rRNA sequencing of fecal samples collected in the third trimester. Rarefaction curves showed that the sequencing depth was sufficient to capture most gene diversity. The results revealed that at the phyla and species levels, the microbiota composition differed between FGR and normal pregnancies (Fig. 1A and B). The α-diversity and β-diversity were also assessed. No significant differences were observed in Chao1, Shannon, and Simpson indices between the two groups (Fig. 1C). The score plot of principal coordinate analysis (PCA) based on unweighted UniFrac distances showed that the gut microbiota of the NP and FGR groups were clearly separated into two clusters (Fig. 1D). At the species level, the relative abundances of *Lactobacillus* (V32) and *Catenibacterium* (V38) were elevated in the FGR group, while the abundances of *Ruminococcaceae* (V22), Bacteroides uniformis (V30), *Mollicutes* RF39 (V52), and Alistipes onderdonkii (V57) decreased (Fig. 1E). Several core species showed significant correlations with fetal measurements based on the Spearman correlation calculated for all participants (Fig. 1F). The abundance of *Catenibacterium* (V38) was inversely correlated with NW, BPD, HC, and FL, and the correlation coefficient for *Lachnospiraceae* (V51) was just opposite. Notably, the abundance of Bacteroides uniformis (V30) was positively associated with fetal HC and BPD. Correlation coefficients of maternal clinical manifestations and core species were also calculated. The abundance of *Ruminococcaceae* (V22) was negatively associated with maternal blood pressure. Maternal total bile acid levels were positively correlated with *Pseudomonas* (V21) (see Fig. S1A in the supplemental material). In addition, we performed a phylogenetic investigation of communities by reconstruction of unobserved states (PICRUSt) analysis. In the FGR group, more Kyoto Encyclopedia of Genes and Genomes (KEGG) pathways were involved mostly in metabolism, including energy metabolism, lipid metabolism, glycan biosynthesis, and amino acid metabolism (Fig. S1B). These results demonstrate altered gut microbial structure and function in patients with FGR.

### Fecal metabolomic alterations in FGR and normal pregnant women.

**FIG 2:**
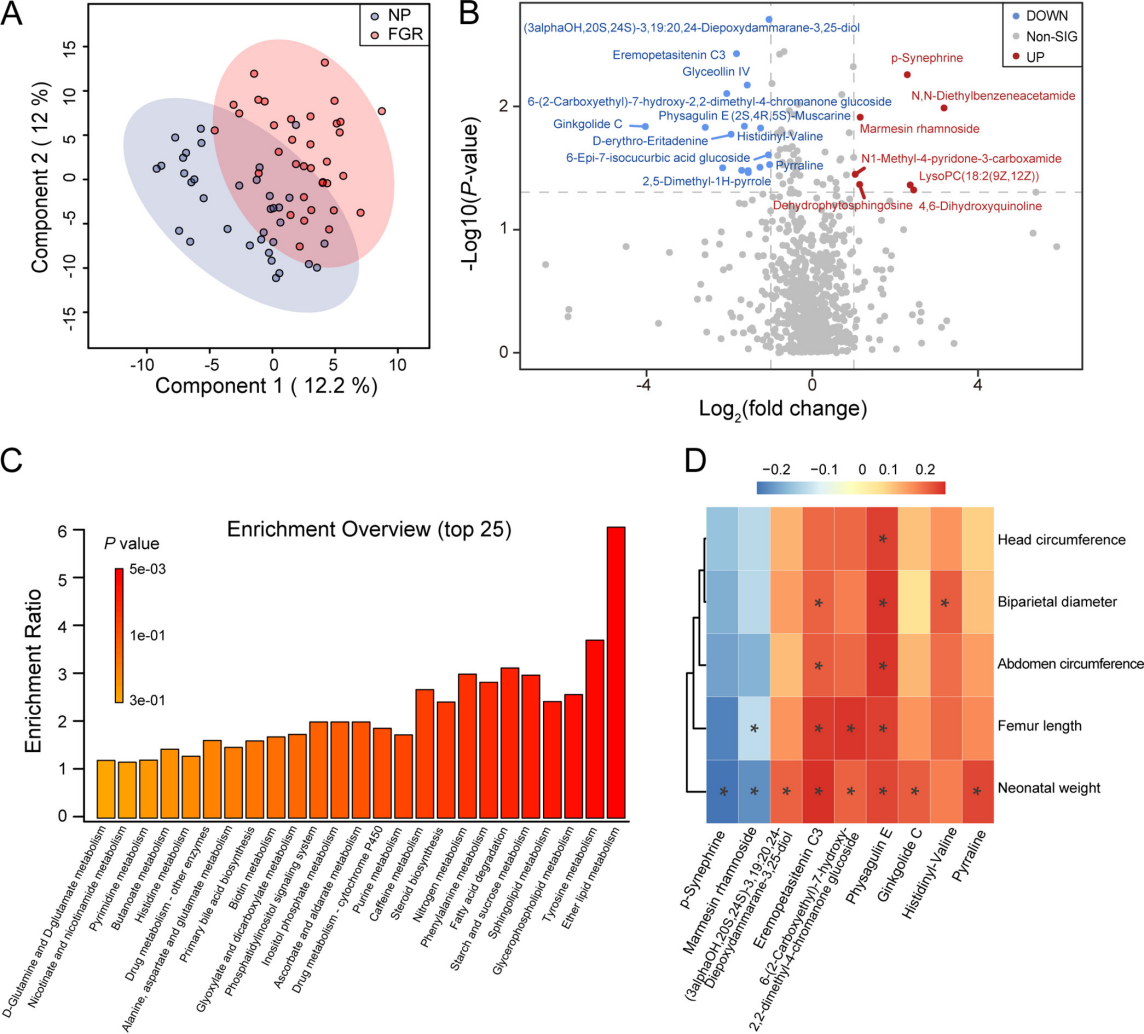
Fecal metabolome profile of patients with FGR and normal pregnant women. (A) PLS-DA analysis of the two groups. (B) The volcano plot of core metabolites, blue for downregulated and red for upregulated in FGR group. (Fold change > 2 and *P *< 0.05). (C) Enriched KEGG pathways involved in FGR group. (D) Correlation heatmap between fetal measurements and the core differential metabolites. Data were processed by Spearman’s correlation test. *, *P *< 0.05; **, *P* < 0.01; ***, *P* < 0.001. NP, *n* = 35; FGR, *n* = 35.

Given the interactions between the gut microbiota and host-microbe cometabolism, we performed liquid chromatography-tandem mass spectrometry (LC-MS/MS) analyses on fecal samples to assess the overall differences in fecal metabolites between FGR (*n* = 35) and normal pregnancies (*n* = 35). In total, 929 features were identified in this study. Partial least-squares projection to latent structures analysis (PLS-DA) revealed that the fecal metabolomic composition of the FGR was distinct from that of the NP group (Fig. 2A). The top 25 most abundant metabolites linked to FGR spanned a broad range of metabolic categories, including amino acids, bile acids, fatty acids, and sphingolipids, among others (see Fig. S2A in the supplemental material). Twenty-three differential metabolites were identified compared to the NP group, including 16 downregulated and 7 upregulated metabolites (Fig. 2B). According to the KEGG pathway database, lipid, amino acid, sphingolipid, fatty acid, and steroid hormone metabolism were enriched in the FGR group (Fig. 2C). Correlation analyses between fecal metabolites and clinical parameters were performed. Physagulin E is closely related to fetal HC, BPD, AC, FL, and NW. Ginkgolide C and pyrraline were also positively associated with NW (Fig. 2D). The metabolites that correlated with maternal clinical manifestations were also clustered. Notably, 1-(beta-d-ribofuranosyl)-1,4-dihydronicotinamide and 4-acetyl-2(3H)-benzoxazolone were negatively associated with systolic and diastolic blood pressure, respectively (Fig. S2B). These results suggest that fecal metabolism is altered in patients with FGR.

### Serum metabolomic alterations in the two groups.

**FIG 3:**
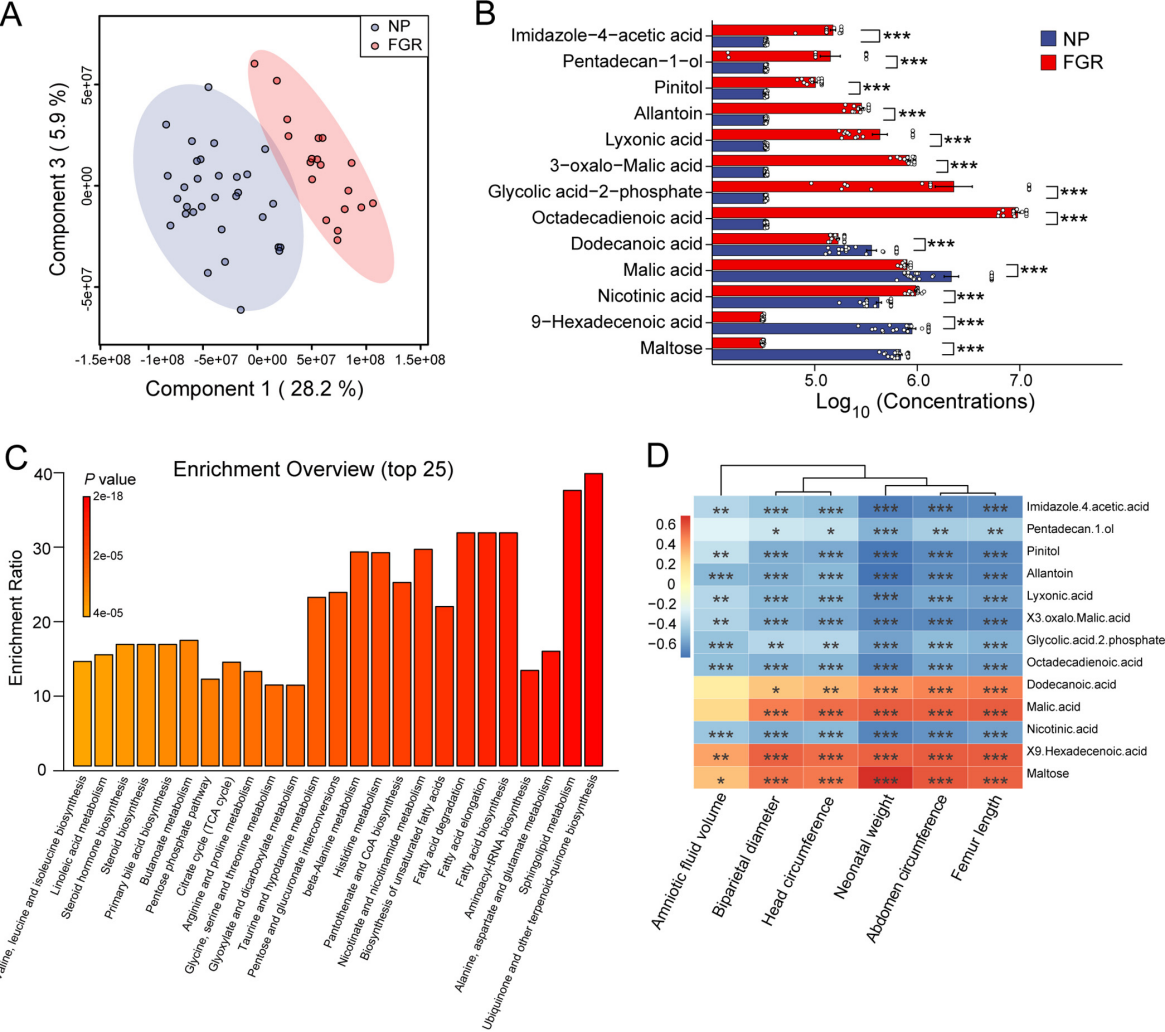
Serum metabolome profile of FGR and NP group. (A) PLS-DA analysis of the two groups. (B) Log_10_(concentrations) of the significantly altered metabolites between NP and FGR groups. ***, padj < 0.001. (C) Enriched KEGG pathways involved in FGR group. (D) Correlation heatmap between fetal measurements and the core differential serum metabolites. Data were processed by Spearman’s correlation test. *, *P* < 0.05; **, *P* < 0.01; ***, *P* < 0.001. NP, *n* = 31; FGR, *n* = 19.

An untargeted metabolome profile was generated using LC-MS/MS to identify the serum metabolome features of patients with FGR and normal pregnancies. For serum metabolism analysis, 50 individuals were recruited from the aforementioned 70 pregnant women, the details of which are presented in Table S1. The PLS-DA score plots showed an apparent separation between the NP and FGR groups (Fig. 3A). Subsequently, we investigated the association between each annotated metabolite and the NP and FGR groups. The top 25 metabolites were clustered, covering amino acids, purines, carboximidic acids, and pyridinecarboxylic acids (see Fig. S3A in the supplemental material). Interestingly, 13 core metabolites were identified as significant. Maltose, malic acid, and 9-hexadecenoic acid were downregulated in the FGR group. In contrast, allantoin, pinitol, nicotinic acid, and lyxonic acid levels were upregulated (Fig. 3B). The KEGG pathway analysis suggested that several functional pathways were enriched in the FGR group. The top 25 pathways analyzed included ubiquinone biosynthesis, sphingolipid metabolism, amino acid metabolism, fatty acid metabolism, and bile acid metabolism (Fig. 3C). Spearman’s correlation analysis was used to identify potential metabolite-clinical variable correlations. Allantoin, a biomarker of oxidative stress (20), was found to be negatively correlated with fetal measurements (AFV, BPD, HC, NW, AC, and FL) and maternal biochemical parameters (aspartate transaminase and alanine transaminase) (Fig. 3D; see also Fig. S3B). In contrast, the malic acid level was positively correlated with fetal HC, BPD, AC, FL, and NW. However, the distinct roles of these compounds in the NP and FGR groups require further study, allowing for potential correlation analysis based on microbiota-metabolite interactions.

### FGR-associated bacterial species contribute to metabolism disruption and link to their clinical profiles.

**FIG 4:**
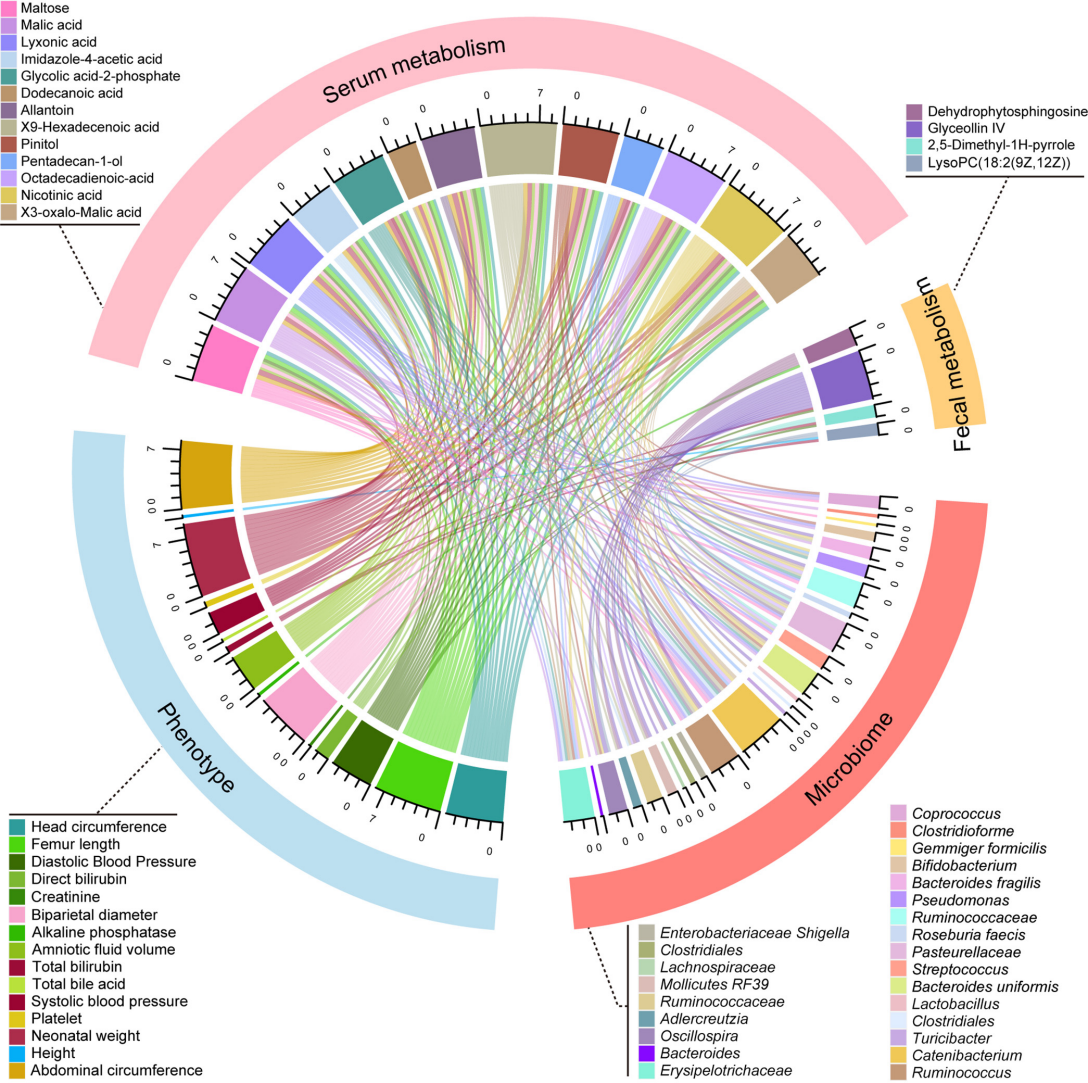
Multi-omics analysis of the altered gut microbiota, fecal and serum metabolites, and maternal-fetal phenotypes. The chord plot showed the pairwise relationships among microbiome, fecal metabolism, serum metabolism, and phenotypes. Correlation tests on all participants for microbiome, fecal metabolism, and phenotypes were performed. As for serum metabolism, corresponding phenotypes and microbiome data were involved in the correlation analysis. Vertices with different colors indicate omics variables, and lines indicate a significant Spearman correlation coefficient at |r| > 0.3 and padj < 0.05. Scales show the sum of correlation coefficients for each variable.

**FIG 5:**
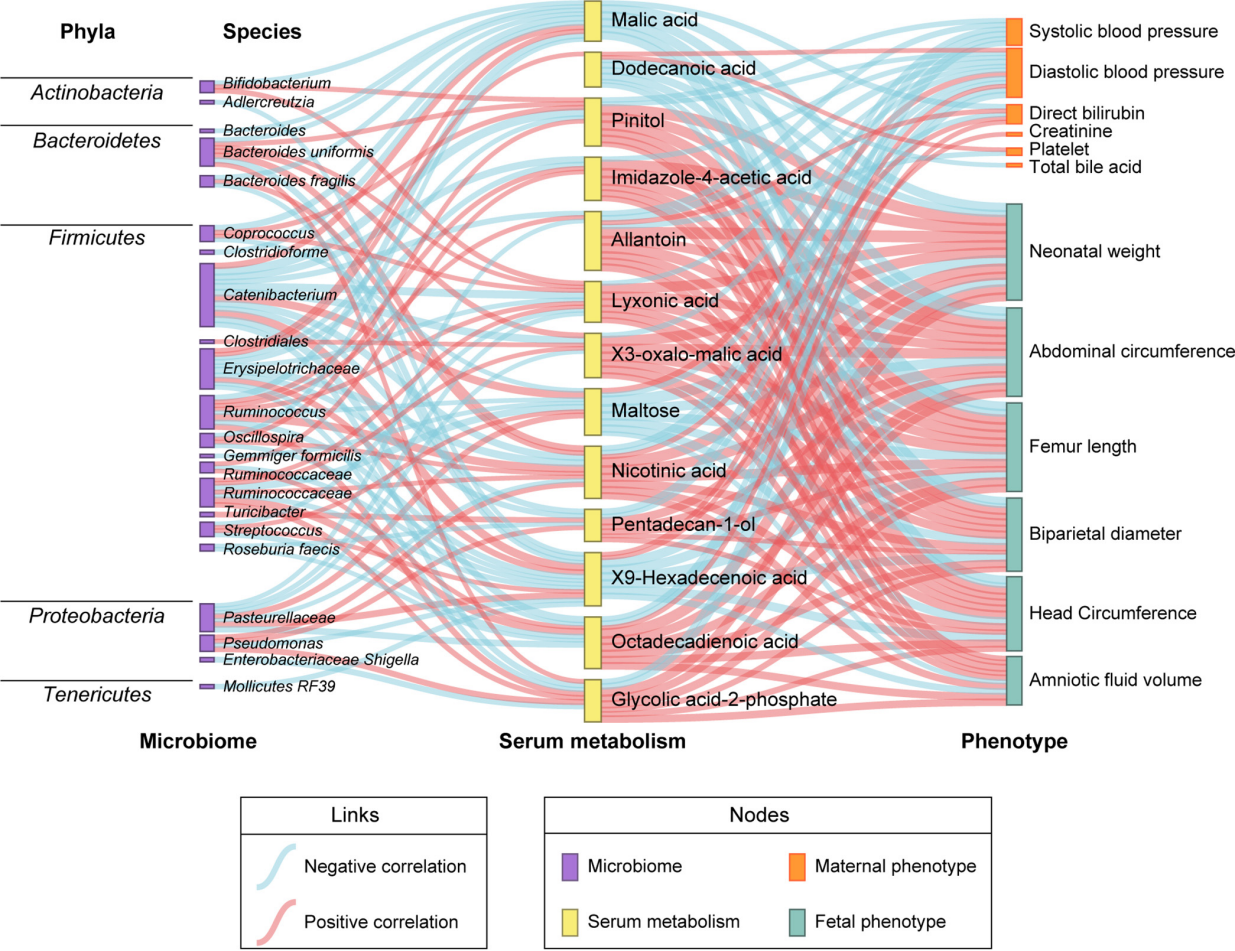
Sankey diagram was depicted to visualize the relationships among microbiome, serum metabolism, and phenotypes in the cohort with 50 participants. The nodes represent omics variables; links in different colors indicate positive and negative correlations, which were calculated by Spearman correlation test, correlation coefficient |r| > 0.3, and padj < 0.05.

We subsequently assessed the correlation between microbiota, metabolites, and clinical phenotypes. An interrelationship network was generated that connected core microbiota species and core metabolites via clinical phenotypes (Fig. 4). Given a Spearman correlation coefficients of 0.3, 25 core bacterial species were significantly associated with 13 serum metabolites and 4 fecal metabolites, which were further related to fetal and maternal measurements. For microbiota and fecal metabolites, *Ruminococcaceae* (V22), *Bacteroid uniformis* (V30), *Lachnospiraceae* (V51), and *Lactobacillus* (V33) were positively correlated with the level of glyceollin IV, which was inversely correlated with systolic blood pressure (SBP) and diastolic blood pressure (DBP). Allantoin, an altered serum metabolite positively related to *Pasteurellaceae* (V29) and *Catenibacterium* (V38), hindered fetal growth distinctively, possibly due to upregulated maternal blood pressure. A similar scenario occurred between *Catenibacterium* (V38), *Enterobacteriaceae Shigella* (V5), and glycolic-acid-2-phosphate. In addition, the elevation of *Erysipelotrichaceae* (V8) in FGR counteracted the promotion effect of dodecanoic acid on fetal growth. Interestingly, these contributing species mainly belong to phylum *Firmicutes*; the rest came predominantly from *Bacteroidetes* and *Proteobacteria* (Fig. 5). Together, these links provide evidence that microbiota dysbiosis plays a critical role in the development of FGR.

### FMT-induced phenotypes of FGR.

**FIG 6:**
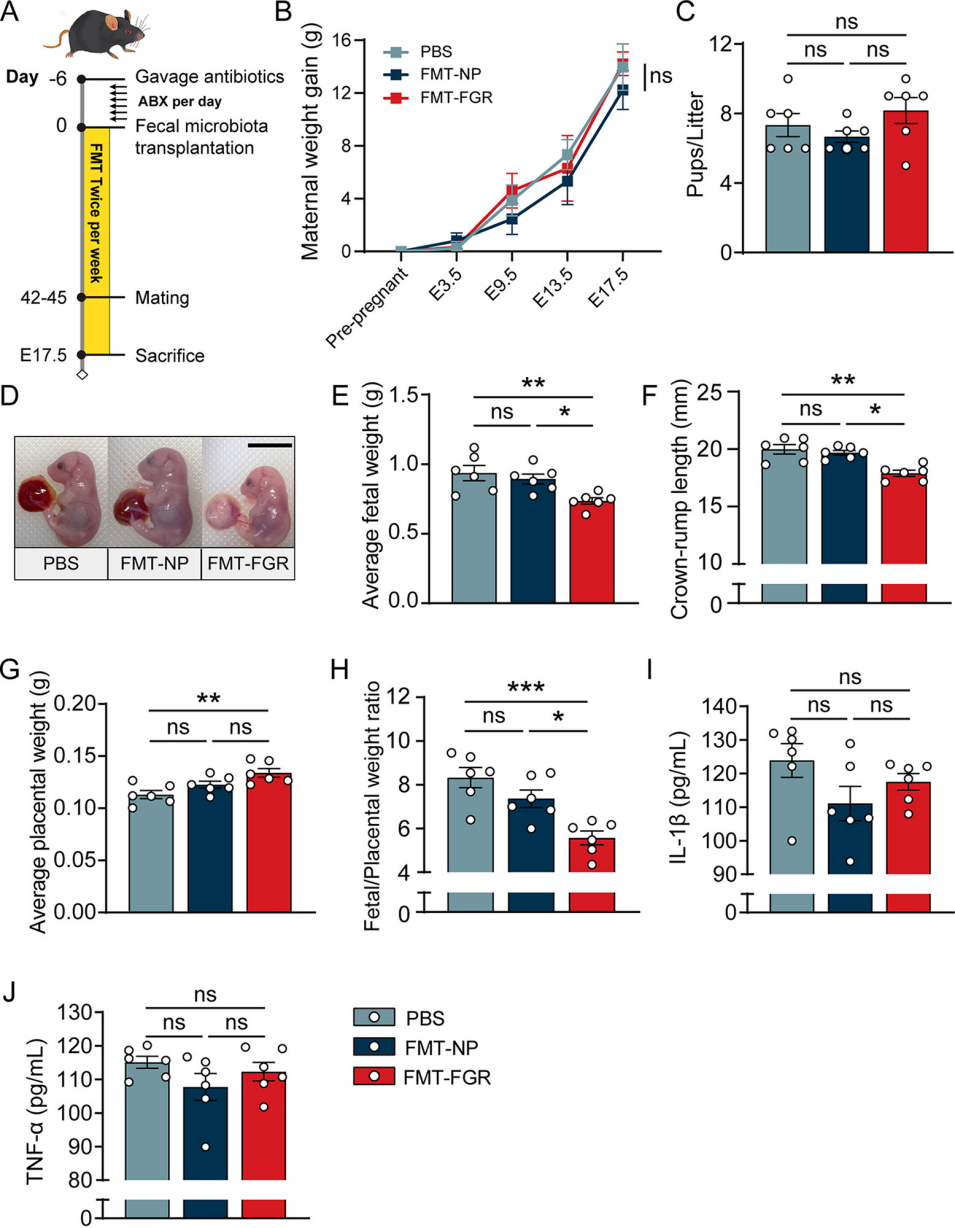
FMT induced FGR phenotypes. (A) Overall timeline of the mouse experiment. (B) Maternal weight gain of each group. (C) Pups/litter of each group. (D) Representative fetus photo of each group. (E) Average fetal weight. (F) Average fetal crown-rump length. (G) Average placental weight. (H) Placental efficiency, calculated as grams fetus/grams placenta. (I) Serum IL-1β level. (J) Serum TNF-α level. For fetal weight, crown-rump length, and placental weight, analyses were performed by taking the average of weights and measurements by litter (5 pups/litter, *n* = 6/group). Data in panel B were presented in mean ± standard deviation (SD) and processed by two-way ANOVA. Data in panels C and E to J were presented as mean ± standard error of the mean (SEM) based on normality tests, and a one-way ANOVA test was conducted followed by a Bonferroni test. *, *P* < 0.05; **, *P* < 0.01; ***, *P* < 0.001. *n* = 6/group.

To validate the impact of microbial communities on FGR, we developed a humanized mouse model by colonizing antibiotic-treated mice with fecal microbiota from FGR patients (FMT-FGR) and normal pregnancies (FMT-NP), as described in Materials and Methods. The overall timeline for the animal experiments with FMT recipients is presented in Fig. 6A. After the 6-week gavage, the body weight gain of the pregnant mice was found to be similar among the groups (Fig. 6B). No differences were observed in the number of pups per litter between each group (Fig. 6C). Compared with mice in the FMT-NP and phosphate-buffered saline (PBS) groups, the FMT-FGR group displayed significantly decreased fetal weight and crown-rump length, whereas the fetal weight of the FMT-NP group and the PBS group showed no difference (Fig. 6D to F). Interestingly, mice that received fecal microbiota from FGR showed significant elevation of placental weight compared to that of the PBS group (Fig. 6G). A reduced fetoplacental weight ratio was observed in the FMT-FGR group, which is assumed to be due to a decreased nutrient net flux per gram placenta, indicating the defects in placental efficiency (Fig. 6H). Enzyme-linked immunosorbent assay (ELISA) was also conducted to evaluate the systemic inflammatory state. The serum interleukin-1β (IL-1β) and tumor necrosis factor-alpha (TNF-α) levels were not significantly altered (Fig. 6I and J). Collectively, these results prove that the administration of microbiota from patients with FGR suppresses fetal growth and reduces placental efficiency.

### FMT impairs the placental function in the FGR mouse.

**FIG 7:**
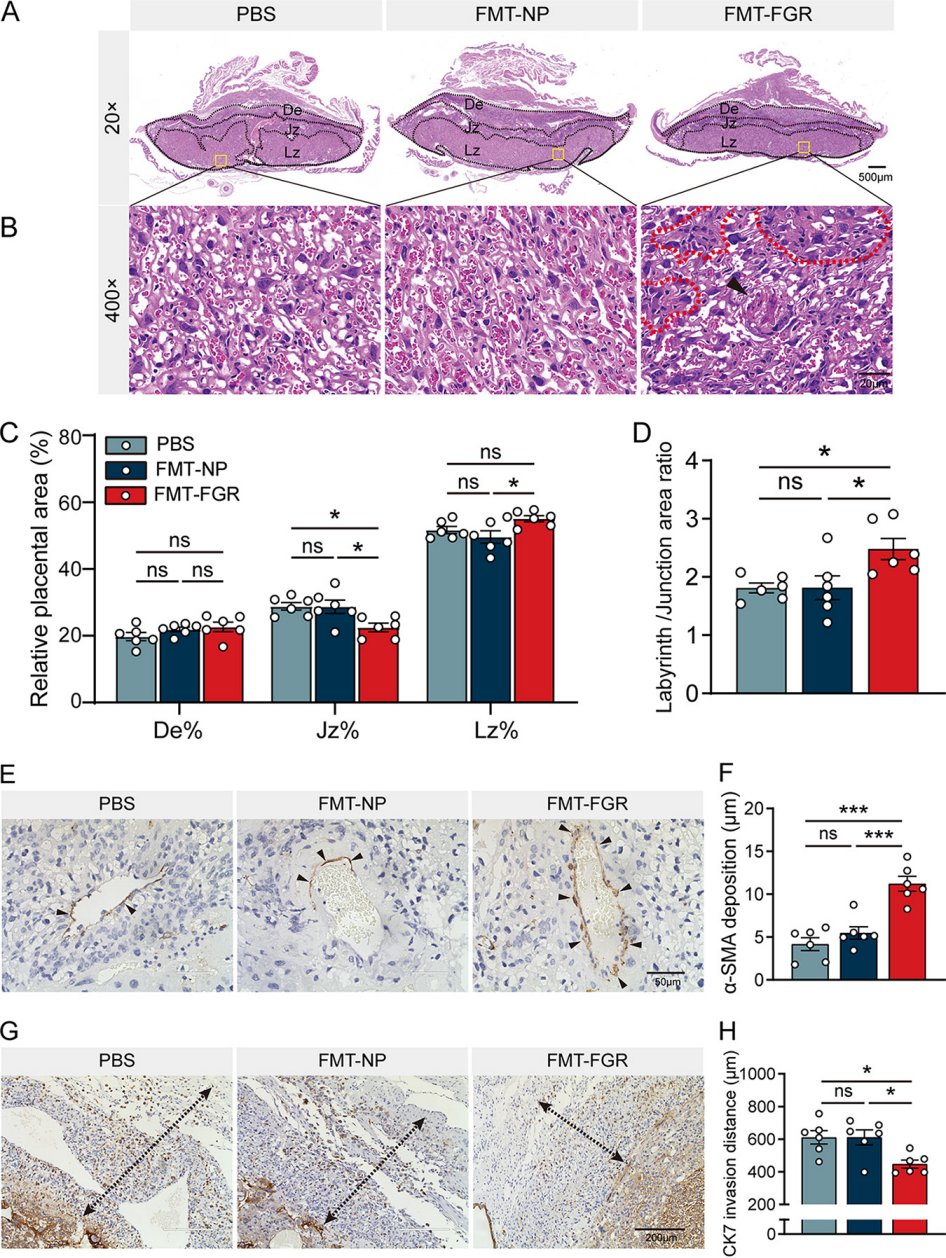
FMT induced placental impairments. (A) Representative images of H&E staining midsagittal placental sections. De, Jz, and Lz areas were marked (magnification, ×20; scale bar = 500 μm). (B) Representative images of H&E staining of the Lz (magnification, ×400; scale bar = 20 μm). The arrowhead indicates the infarction lesion in the Lz, and the dotted lines indicate swollen tissues and restricted maternal sinusoidal blood space. (C) The areas of De, Jz, and Lz were expressed as the percentage of the total placental area. (D) The ratio between the Lz/Jz. (E) Representative images of α-SMA immunohistochemistry staining (scale bar = 50 μm). The arrowheads denote α-SMA-positive cells. (F) Quantification of α-SMA-positive cells surrounding decidual spiral arteries. (G) Representative images of CK-7 immunohistochemistry staining. The dotted lines with arrowheads show the invasive depth of CK-7-positive cells into the De (scale bar = 200 μm). (H) Maximum distance of CK-7-positive cell invasion into the De (*n* = 6/group). Data were presented as mean ± SEM and processed by one-way ANOVA. *, *P* < 0.05; **, *P* < 0.01; ***, *P* < 0.001. *n* = 6/group. FMT, fecal microbiota transplantation; De, decidua; Jz, junctional zone; Lz, labyrinth zone; α-SMA, alpha-smooth muscle actin; CK-7, cytokeratin-7; ns, not significant.

To further determine the cause of the defective placental function, we performed hematoxylin and eosin (H&E) staining on mouse placenta, which was comprised of three distinct zones. They are maternally derived decidua (De) and the conceptus-derived junctional zone (Jz) and labyrinth zones (Lz). To assess the placental development, the percentage of area in the placental layers has been assessed (21). The percentage of Lz was higher in the FMT-FGR group than that in the FMT-NP group, whereas the percentage of Jz was significantly reduced in the FMT-FGR group compared with other groups; no difference in De was noted among the three groups (Fig. 7A and C). In addition, we also analyzed the ratio of labyrinth to junctional zone, which was correspondingly increased in the FMT-FGR group (Fig. 7D). In line with these findings, morphological differences in the Lz were evident in the FMT-FGR group; these placentas exhibited infarction lesions surrounded by swollen tissues. However, the FMT-NP group resembled the controls (Fig. 7B). Immunohistochemistry (IHC) was performed to evaluate the placentation. The expression of α-smooth muscle actin (α-SMA) was markedly higher in the FMT-FGR group according to IHC (Fig. 7E and F), indicating inadequate reconstruction of the uterine spiral arteries. Placenta from the FMT-FGR group also showed limited cytotrophoblast invasion compared with the others, while no significant differences were noted between the PBS and FMT-NP groups (Fig. 7G and H). Additionally, evidence has shown that fetal growth relies on the capacity of the placenta to supply nutrients from the mother (22). Therefore, we have detected the relative expression of placental transporters using reverse transcriptase quantitative PCR (RT-qPCR). As shown in Fig. S4A to D in the supplemental material, no significant difference was observed in the expression levels of markers associated with glucose and amino acid transportation (*Glut1*, *Glut3*, *Slc38a1*, and *Slc38a2*) among the three groups. While the relative expression of the fatty acid transporter (*Cd36*) showed a non-statistically significant downward trend, a notable decrease of *Fabp3* was presented in the FMT-FGR group compared to the placenta of the PBS group (Fig. S4E and F). These results demonstrate that the unfavorable uterine environment and related phenotypes were triggered mainly by FGR-specific microbial signatures.

## DISCUSSION

In the present study, we reported a multi-omics analysis based on the gut microbiome and fecal and serum metabolome in patients with FGR and normal pregnancies. Our integrated analysis revealed that the characteristics of the taxa, metabolites, and pathways involved were widely correlated. These findings that gut dysbiosis can induce FGR was validated in an FMT mouse model, providing a new perspective on the pathogenesis of this disease.

Alterations in the microbial community composition and taxa abundance were observed in patients with FGR. Many altered differential taxa and metabolites have been identified in patients with FGR, which may indicate a general mechanism for FGR. A bloom in populations of unassigned species in the genus *Lactobacillus* and the family *Erysipelotrichaceae* was observed in FGR patients. We also found a significant negative correlation between the genus *Catenibacterium*, which belongs to the family *Erysipelotrichaceae*, and fetal growth measures, including NW, BPD, HC, and FL. Interestingly, both *Lactobacillus* and *Erysipelotrichaceae* have been identified as the microbial signatures in patients with metabolic syndrome and nonalcoholic fatty liver disease; such diseases share the same etiology of systemic low-grade inflammation (23–25). Emerging evidence indicates that aberrant maternal inflammation may contribute to defects in trophoblast function and placental dysfunction, which further causes FGR (26). It has been reported that SCFAs may regulate intestinal immunity via the production of interleukin-22 by innate lymphoid cells and CD4^+^ T cells (27). In a recent study, supplementation of SCFAs, particularly propionate, was shown to reverse the reduction in birth weight in hypoxia-induced FGR (28). In line with these findings, our data showed that the abundance of SCFA producers *Ruminococcaceae*, Bacteroides uniformis, and Alistipes onderdonkii was relatively lower, coupled with the involvement of butanoate metabolism in patients with FGR (29, 30). The reduction in Bacteroides uniformis and Alistipes onderdonkii may directly inhibit succinate synthesis, which is propionate’s major precursor (29, 30). In addition, the relative abundance of uncharacterized species belonging to *Ruminococcus* and *Lachnospiraceae*, the two predominant families of butyrogenic *Firmicutes*, displayed a strong positive association with NW, as well as BPD, HC, and FL, which may partly be due to their immunomodulatory properties (31, 32).

Through the FMT mouse model, FGR-specific microbiota can induce abnormal fetal growth and decreased placental efficiency, which is commonly used as a retrospective indication to evaluate fetal development. It is increasingly recognized that failure to transform uteroplacental spiral arteries and poor trophoblast cell invasion underpin a range of pregnancy complications, such as FGR (8, 33). Thus, we analyzed α-SMA, a marker of vascular smooth muscle cells, and cytokeratin 7, a marker of trophoblasts in the mouse placentas from each group, in addition to H&E staining. Although the FMT-FGR group exhibited a larger placenta size, impairments and poor placentation were evident, which may offset the compensatory placental enlargement. However, the whole fecal materials administered to mice include bacteria, fungi, viruses, phages, proteins, and metabolites (34), which are the key factors that need further identification.

Metabolic profiles of feces and serum revealed remarkable alterations in response to FGR. Fecal metabolites can reflect the status of the gut microbiota and the relationship between the symbiotic flora and the host. Here, we identified 23 differential metabolites in fecal metabolomic analysis, such as p-synephrine, N1-methyl-4-pyridone-3-carboxamide, ginkgolide C, and other metabolites that have not been fully studied. Among them, p-synephrine and N1-methyl-4-pyridone-3-carboxamide were upregulated, while ginkgolide C was decreased in FGR patients. Consistent with our findings, N1-methyl-4-pyridone-3-carboxamide has been detected in serum and amniotic fluid samples in gestational diabetes mellitus (GDM) patients (35). Furthermore, a negative correlation between N1-methyl-4-pyridone-3-carboxamide and fetal weight has been reported in those patients involved in nicotinate and nicotinamide metabolism (35). A recent study showed that ginkgolide C might promote intestinal barrier function and exert anti-inflammatory effects by inhibiting the nuclear factor kappa-light-enhancer of activated B cells (NF-κB) and mitogen-activated protein kinase (MAPK) pathways in a dextran sulfate sodium-induced colitis model (36). The current study showed that the concentration of ginkgolide C was positively associated with neonatal weight, suggesting its potential to serve as a biomarker for FGR. Other metabolites, such as physagulin E, eremopetasitenin C,3 and marmesin rhamnoside, are closely correlated with fetal weight and remain to be further illustrated. We also identified 13 differential metabolites in serum samples that were strongly related to NW, BPD, HC, AC, FL, and AFV. For example, an increase in allantoin, a metabolite of uric acid, has also been reported in patients with obesity and sepsis, indicating oxidative stress and inflammatory status in FGR patients, which still needs further validation (37, 38). In addition, dodecanoic acid downregulates proinflammatory factors and attenuates liver inflammation by reducing Toll-like receptor 4 (TLR-4)/NF-κB pathway expression in a lipopolysaccharide (LPS)-induced murine model (39). Despite these discrepancies, metabolomic enrichment analysis of the feces and serum showed similar metabolic pathways, including nicotinate and nicotinamide metabolism; butanoate metabolism; histidine metabolism; and alanine, aspartate, and glutamate metabolism. Others include primary bile acid biosynthesis, glyoxylate and dicarboxylate metabolism, steroid biosynthesis, fatty acid degradation, sphingolipid metabolism, and tyrosine metabolism. However, there appeared to be no overlapping altered metabolites between feces and serum specimens. We acknowledge that bias may occur owing to the unmatched sample size between fecal microbiota analysis and serum metabolism analysis. Although the smaller sample size in serum metabolism analysis may result in a lower statistical power, attempts to mitigate this effect have been made using appropriate statistical analyses.

Of note, an integrated cross-omics framework was conducted to better understand the links between the gut microbiome and metabolome, showing that many metabolic features have substantial links to microbial signatures and clinical features. It has been proposed that elevated maternal blood pressure contributes to reduced fetal growth even within the normal range (40, 41), which corroborates our statement that metabolites including allantoin and glycolic-acid-2-phosphate serve as mediums of microbial features to FGR phenotypes. Additionally, recent studies on the microbiota-gut-brain axis indicated the interaction between gut microbiota and human neurophysiology and mental health (42, 43). Over 5% of pregnant women suffer from panic disorder, and about 10% report anxiety symptoms (44). In this study, patients diagnosed with FGR may have a higher risk for mental disorders than patients in the NP group. Previous study has shown that prenatal anxiety was specifically related to maternal gut dysbiosis, whereas variable information (i.e., stressful events) was not incorporated in the study (45). Therefore, future large cohorts are needed to investigate the associations between fetal growth, gut microbiota, and maternal psychosocial stress.

In conclusion, our study demonstrates gut dysbiosis in patients with FGR and is the first attempt to reveal potential links between gut microbiota and metabolites from feces and serum, providing new insight into the pathogenesis of FGR.

## MATERIALS AND METHODS

### Study participants.

Ethical approval was granted by the Ethics Committee of the Nanfang Hospital (NFEC-2020155). Women diagnosed with FGR and normal pregnant women were recruited in the third trimester from November 2020 to March 2021 in the Department of Obstetrics of the Nanfang Hospital, Southern Medical University, China. According to the birth weight curve published by the National Institute of Child Health and Human Development (46), the inclusion criteria for the FGR group were as follows: an estimated fetal weight or abdominal circumference less than the 10th percentile for gestational age (1) and placental disorders or umbilical cord abnormalities by postnatal confirmation. Women with the following conditions were excluded: (i) administration of any antibiotic or probiotic treatments 1 month before fecal sample collection; (ii) diseases that may affect the gut microbiota, such as inflammatory bowel disease, irritable bowel syndrome, and diarrhea; and (iii) other metabolic diseases or obstetric complications. Fecal and serum samples from the same volunteer were collected on the same day. Serum samples were obtained from each participant on the same day as fecal samples with informed consent and were centrifuged at 3,000 rpm at 4°C for 10 min. The supernatants and fecal samples were stored at −80°C before further processing.

### Fecal DNA extraction and 16S rRNA sequencing.

Fecal DNA was extracted using QIAamp PowerFecal Pro DNA kit (Catalog no. 51804; Qiagen, Inc., Germany) following the manufacturer’s protocol. The quantity and quality of DNA were measured using a Biodropsis BD-1000 spectrophotometer (OSTC, Inc., China) and agarose gel electrophoresis, respectively. PCR amplification of the bacterial 16S rRNA gene V4 region (F515/R806) was performed according to previously described protocols (47). All PCR amplicons were mixed and sequenced using the Illumina Nova 6000 platform (2 × 250 bp, paired-end) following the manufacturers protocol. All DNA samples from 70 fecal samples were subjected to the same sequencing run.

### Gut microbiota analysis.

After sequencing, the raw sequences were processed using Quantitative Insights into Microbial Ecology 2 (QIIME2) with a standard pipeline (48). Paired-end reads were assigned to samples based on their unique barcodes. The high-quality amplicon sequence variants (ASVs) were obtained by Divisive Amplicon Denoising Algorithm 2 (DADA2) (49). Raw ASV data counts were transformed into relative abundance. Those whose relative abundance was less than 0.001 or the attendance rate was less than 70% and microbial data was filtered for core bacterial taxa. Taxonomic profiling was performed using the Greengenes database and transformed into relative abundances at the phylum, class, order, family, genus, and species levels. Core bacterial analysis, including α-diversity, β-diversity, and structure plots were performed using the R package EasyMicroPlot (50).

### Sample preparation for metabolomics study and data analysis.

We collected 70 fecal samples (*n* = 35 for NP and *n* = 35 for FGR) and 50 serum samples (*n* = 31 for NP and *n* = 19 for FGR) for metabolomic analysis. The fecal (51) and serum metabolite extraction (52) were conducted based on previously published methods. Briefly, the fecal samples were quantified, homogenized, and centrifuged. The supernatants were used for LC-MS/MS analysis on an ultrahigh performance liquid chromatography (UHPLC) system (Vanquish; Thermo Fisher Scientific) with an ultra-performance liquid chromatography ethylene-bridge hybrid (UPLC BEH) Amide column coupled to a Q Extractive HF-X mass spectrometer (Orbitrap MS; Thermo), according to the manufacturer’s instructions. Samples were analyzed in both positive and negative ion modes. The acquired raw data was transformed into mzXML format. Subsequently, it was processed with peak identification, peak alignment, peak extraction, retention time correction, and peak integration, as previously described (53). The downstream analyses were performed using the R package MetaboAnalystR 5 (54).

### Animal model.

The mouse experiments were approved by the Ethics Committee of Southern Medical University (no. L2020098). Six-week-old specific-pathogen-free C57BL/6J mice were purchased from Guangdong Medical Laboratory Animal Center and were bred at the Experimental Animal Research Center at Southern Medical University. All mice were maintained in a temperature-controlled specific-pathogen-free facility on a 12-h light-dark schedule and had free access to autoclaved food and water. Fecal microbiota transplantation (FMT) was performed using a previously described method (15). Briefly, female mice received a cocktail of vancomycin (100 mg/kg), neomycin sulfate (200 mg/kg), metronidazole (200 mg/kg), and ampicillin (200 mg/kg) via oral gavage once daily for five consecutive days. After a washout period, the animals received fecal supernatant mixtures from donors. We randomly selected three patients with FGR in the FMT-FGR group and three normal pregnant women as FMT-NP donors. The control group was administered phosphate-buffered saline (PBS). Mice were inoculated with fecal supernatant mixtures twice a week. After 6 weeks, female mice were mated with untreated wild-type C57BL/6J male mice at a 2:1 to 3:1 ratio overnight. Pregnancies were dated by the presence of a vaginal plug (embryonic day [E] 0.5). On E17.5, mice were anesthetized with pentobarbital sodium (45 mg/kg, intraperitoneally [i.p.]). Blood samples were collected from the inferior vena cava and were centrifuged. The supernatants were stored in −80°C until analysis. The pups and placentas were dissected from the uterus and weighed. The crown-rump length was measured using a vernier caliper.

### Morphology and histology.

Mouse placentas were harvested and fixed in 4% paraformaldehyde. Subsequently, they were rinsed in PBS, transferred in ethanol (70%), and processed into paraffin-embedded tissue blocks. Tissue sections of placenta (4 μm) were selected for hematoxylin and eosin (H&E) staining and analyzed by microscopy. Structural analysis of the labyrinth was performed as previously described (55). Immunohistochemistry was performed using primary antibodies with α-SMA, cytokeratin-7 (ab124964, dilution 1:100; ab181598, dilution 1:8000; Abcam, Cambridge, UK) according to the manufacturer’s protocol. Briefly, sections were deparaffinized and rehydrated. After antigen retrieval, the sections were blocked with 1% bovine serum albumin at room temperature for 1 h. Subsequently, they were incubated with the antibodies at 4°C overnight. The sections were then incubated with a secondary antibody and treated with 3,3′-diaminobenzidine solution for microscopic imaging.

### Enzyme-linked immunosorbent assay.

The enzyme-linked immunosorbent assay (ELISA) was performed on mouse serum samples using ELISA kits (Bioswamp, Wuhan, China). The proinflammatory indicators interleukin-1β (IL-1β) and tumor necrosis factor-alpha (TNF-α) were detected according to the manufacturer’s instructions.

### Total RNA extraction and RT-qPCR analysis.

Total RNA from placenta was extracted using TRIzol reagent (Invitrogen, USA). Reverse transcription was performed using a commercial kit (CoWin Biosciences, Taizhou, China) according to the manufacturer’s protocols. For gene expression analysis, RT-qPCR was conducted on a LightCycler 96 instrument (Roche) using ChamQ SYBR qPCR master mix (Vazyme, Nanjing, China) following the manufacturer’s instructions. The primer sequences for the target genes were as follows: *Glut1* forward, 5′-GCTGTGCTTATGGGCTTCTC-3′, reverse, 5′-ACACCTGGGCAATAAGGATG-3′; *Glut3* forward, 5′-GGAGGAGAACCCTGCATATGATA-3′, reverse, 5′-TGGCTTCATAGTCATCCTT- -TAGTAAC-3′; *Slc38a1* forward, 5′-GTCAGCAACGACTCTAATGACTT-3′, reverse, 5′-GGAATAT- -ACTCGTCGCATTTCCT-3′; *Slc38a2* forward, 5′-ATGAAGAAGACCGAAATGGGAAG-3′, reverse, 5′-TGGTGGGGTATGAGTAGTTGAA-3′; *CD36* forward, 5′-CAGTGCAGAAACAATGGTTGTCT-3′, reverse, 5′-TGACATTTGCAGGTCTATCTACG-3′; *Fabp3* forward, 5′-CTGTCACCTCGTCGAA- -CTCT-3′, reverse, 5′-TTTGTCGGTACCTGGAAGCT-3′; *18S* forward, 5′-AGTCCCTGCCCTTTGT- -ACACA-3′, reverse, 5′-CGATCCGAGGGCCTCACTA-3′. *18S* RNA were used as endogenous reporter, and fold changes in gene expression levels were quantified using the 2^−(ΔΔ^*^CT^*^)^ method with normalization to the corresponding reference gene.

### Statistical analysis.

The algorithms were chosen appropriately according to different data types. The normality of data was assessed using the Shapiro-Wilk test. For normally distributed data, unpaired Student's *t* test was performed to compare the differences between two groups. One-way analysis of variance (ANOVA) and two-way ANOVA, followed by Bonferroni’s *post hoc* test, were conducted to compare the groups. For nonnormal distributed variables, the Mann-Whitney U test was conducted to compare two groups for continuous variables, and the Kruskal-Wallis test was performed to compare medians among groups followed by Dunn’s test. The chi-square test was used to compare the categorical variables. Correlations between taxa or metabolites and phenotypes were calculated using Spearman’s correlation test. In the mouse model, fetal weight and placental weight for each group per litter were assessed. Five fetuses and respective placentas per dam were randomly collected on E17.5 to calculate litter averages of fetal and placental weights (56). Placental efficiency was obtained by normalizing the fetal weight to its corresponding placental weight. Statistical analyses were performed and plotted using GraphPad Prism v8.0.2 software (GraphPad Software, San Diego, CA, USA), R v4.1.2 (R Foundation for Statistical Computing, Vienna, Austria; http://www.R-project.org/), and Microsoft Excel (Microsoft, Seattle, WA, USA).

### Data availability.

The raw sequencing data was deposited in the NMDC database under accession number NMDC10018388.
