# Supplementary material for: Alterations in the Gut Microbiome and Metabolisms in Pregnancies with Fetal Growth Restriction
Source: Microbiol Spectr. 2023 May 18;11(3):e00076-23. doi: 10.1128/spectrum.00076-23 (PMC10269609; doi:10.1128/spectrum.00076-23)
Supplement: Supplemental file 1 — Supplemental material. Download spectrum.00076-23-s0001.pdf, PDF file, 1.0 MB [file spectrum.00076-23-s0001.pdf]

Alterations in the gut microbiome and metabolisms in pregnancies with fetal growth restriction

Running title: Gut dysbiosis and metabolic disorders in FGR

Zixin Tao[1#], Yun Chen[1#], Fang He[2], Jiawei Tang[3], Limei Zhan[4], Haoyue Hu[1], Ziling Ding[1], Shenghang Ruan[5], Yutao Chen[3], Beier Chen[3], Yan Wang[1], Xiaoling Guo[4], Liwei Xie[6*], Mei Zhong[1*], Qitao Huang[7*]

[1]Department of Obstetrics and Gynecology, Nanfang Hospital, Southern Medical University, Guangzhou, Guangdong, China

[2]Department of Obstetrics and Gynecology, The Third Affiliated Hospital of Guangzhou Medical University, Guangzhou, Guangdong, China

[3]The Second School of Clinical Medicine, Southern Medical University, Guangzhou, Guangdong, China

[4]Foshan Women and Children Hospital Affiliated to Southern Medical University, Foshan, Guangdong, China

[5]The First School of Clinical Medicine, Southern Medical University, Guangzhou, Guangdong, China

[6]State Key Laboratory of Applied Microbiology Southern China, Guangdong Provincial Key Laboratory of Microbial Culture Collection and Application, Guangdong Open Laboratory of Applied Microbiology, Institute of Microbiology, Guangdong Academy of Sciences, Guangzhou, Guangdong, China

[7]Department of Obstetrics and Gynecology, The First People's Hospital of Foshan, Foshan, Guangdong, China

***Correspondence:**

Professor Qitao Huang, Department of Obstetrics and Gynecology, The First People's Hospital of Foshan, Foshan, Guangdong, China, 528000; 15920454515@163.com

Professor Mei Zhong, Department of Obstetrics and Gynecology, Nanfang Hospital, Southern Medical University, Guangzhou, Guangdong, China, 510515; zhongmei@smu.edu.cn

Professor Liwei Xie, State Key Laboratory of Applied Microbiology Southern China, Guangdong Provincial Key Laboratory of Microbial Culture Collection and Application, Guangdong Open Laboratory of Applied Microbiology, Institute of Microbiology, Guangdong Academy of Sciences, Guangzhou, Guangdong, China, 510070; xielw@gdim.cn

[#] These authors contributed equally to this work.

Supplementary materials

Supplementary Table 1

	NP Group (n=31)	FGR Group (n=19)	*P*
**Maternal features**			
Age (y)	30.10±4.99	28.60±3.53	0.3322
Gestational Age (w)	39.80±1.18	37.20±1.77	<0.001
BMI (kg/m^2)	26.80±3.00	26.30±3.97	0.6154
SBP (mmHg)	117 (112-124)	124 (120-138)	0.0024
DBP (mmHg)	68.90±7.97	77.42±8.25	0.0009
PLT ($\times 10^9$/L)	229.6±59.76	250.8±54.14	0.2025
ALT (U/L)	10.27±2.97	11.87±4.98	0.2157
AST (U/L)	14.9 (12.8-17.8)	17.5 (13.2-21.8)	0.1647
ALP (U/L)	158 (131-181)	139 (117-179)	0.4662
ALB (g/L)	36.36±2.02	35.23±3.83	0.2462
TBA (µmol/L)	3.3 (2.4-5.8)	3.1 (2.6-4.6)	0.6807
TBil (µmol/L)	8.0 (7.0-9.7)	6.6 (5.5-8.2)	0.0031
DBil (µmol/L)	1.4 (1.1-1.9)	1.1 (0.8-1.5)	0.0227
Cr (µmol/L)	45.0 (42.0-49.0)	51.0 (40.0-56.0)	0.3182
**Fetal features**			
NW (g)	3310±301.6	2151±239.2	<0.0001
BPD (mm)	92.0 (86.0-95.0)	83.0 (75.0-87.0)	<0.0001
HC (mm)	328 (317-334)	296 (274-311)	<0.0001
AC (mm)	334 (325-345)	286 (269-303)	<0.0001
FL (mm)	72 (69-73)	63 (58-67)	<0.0001
AFV (mm)	48.8±11.6	36.7±9.0	0.0001

Data was assessed for normality using the Shapiro-Wilk normality test, and was presented as mean ± SD or median (interquartile range). For normally distributed data, Student's *t*-test with Welch's correction was performed between two groups. For non-normal distributed data, Mann–Whitney U test was performed between two groups. BMI, body mass index; G, gravidity; P, parity; SBP, systolic blood pressure; DBP, diastolic blood pressure; PLT, platelet; ALT, alanine aminotransferase; AST, aspartate aminotransferase; ALP, alkaline phosphatase; ALB, albumin; TBil, total bilirubin; DBil, direct bilirubin; Cr, creatinine; NW, neonatal weight; BPD, biparietal diameter; HC, head circumference; AC, abdominal circumference; FL, femur length; AFV, amniotic fluid volume.

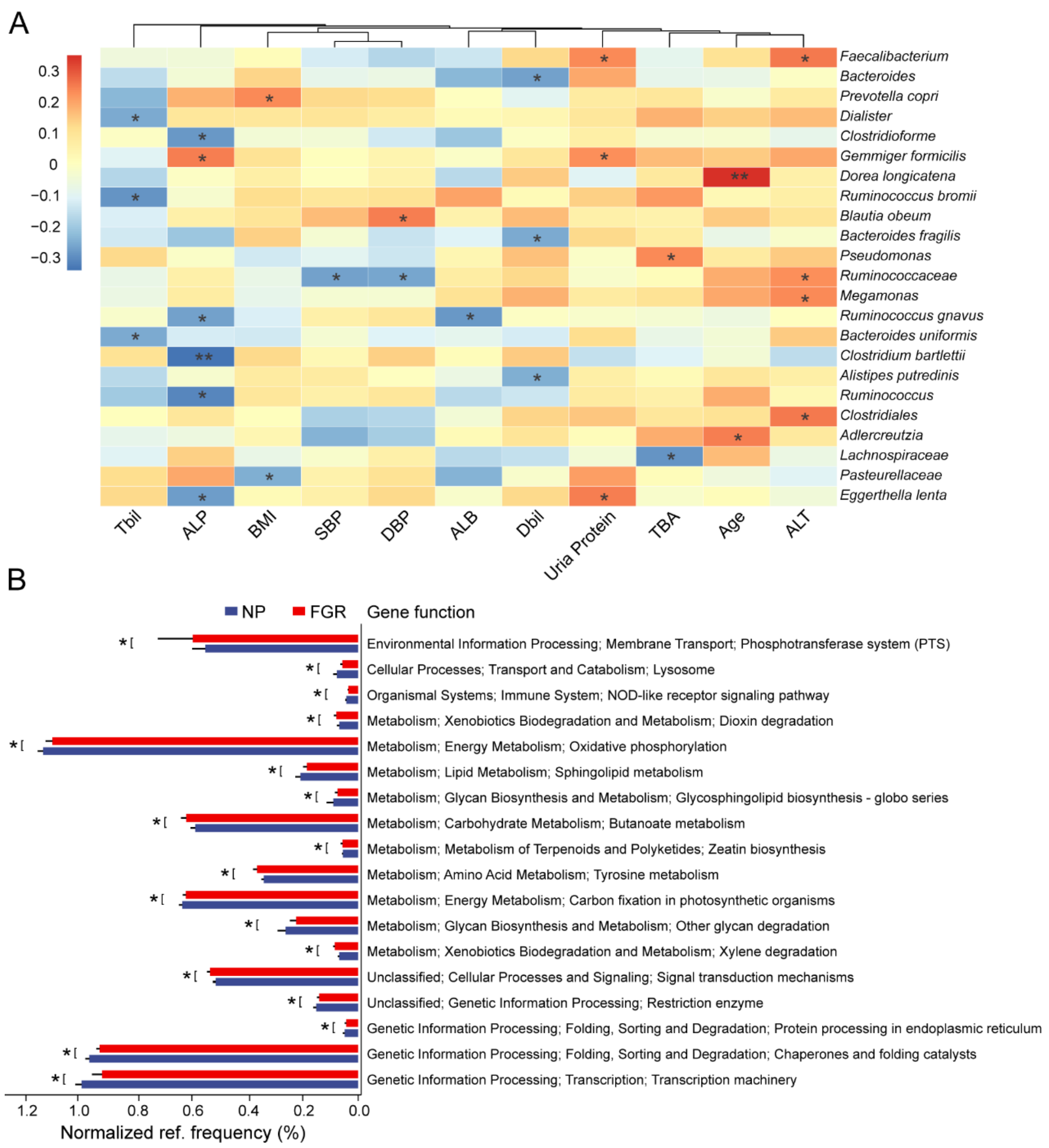

Supplementary Figure 1 (A) Correlation heatmap of maternal gut microbiota and maternal clinical manifestations. Data was processed by Spearman's correlation test. **(B)** KEGG pathways predicted by PICRUSt analysis. (* $P < 0.05$, ** $P < 0.01$, *** $P < 0.001$. NP, n = 35; FGR, n = 35)

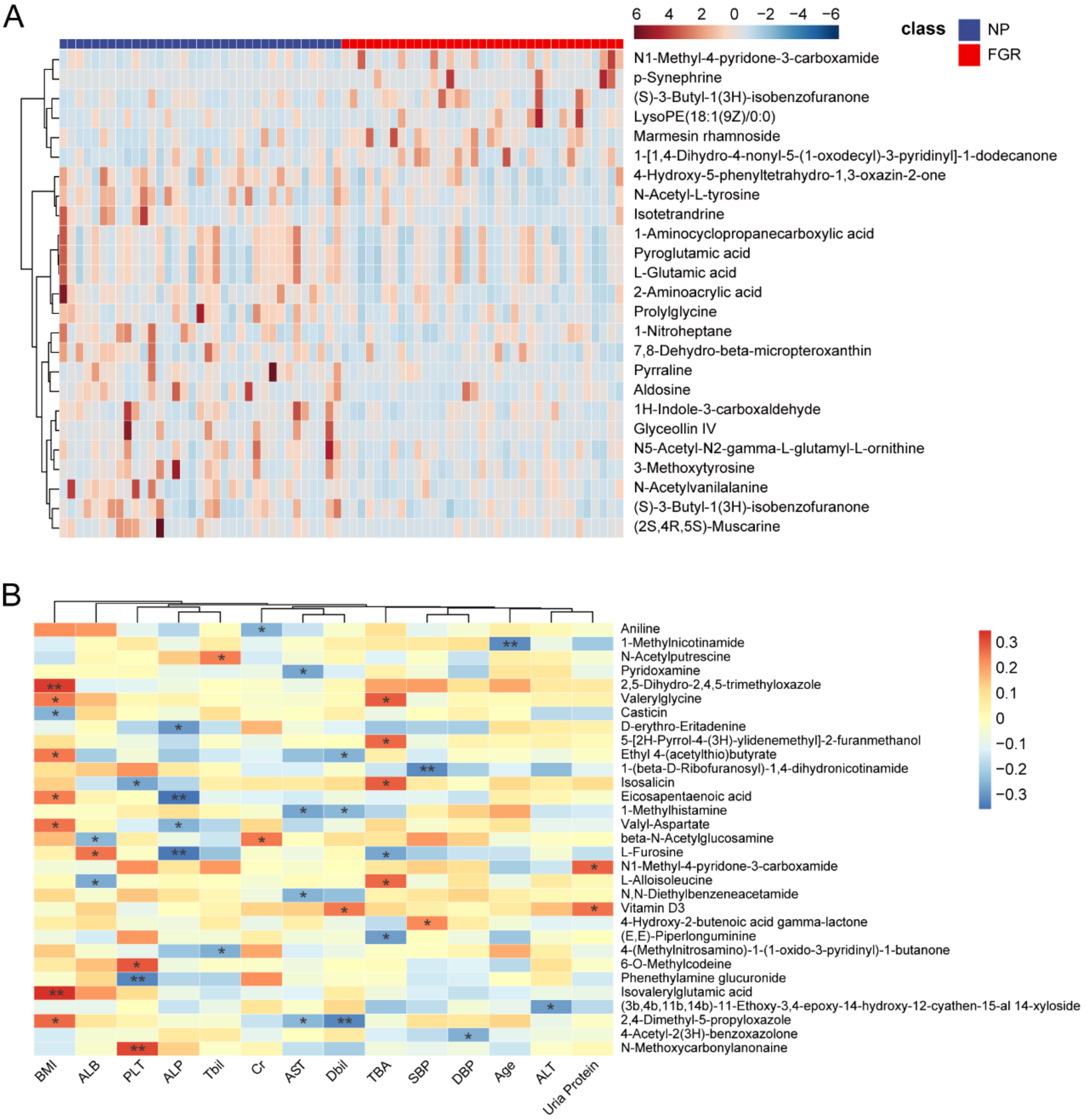

Supplementary Figure 2 (A) The top 25 abundant metabolites. **(B)** Correlation heatmap of maternal gut metabolites and maternal clinical manifestations. Data was processed by Spearman's correlation test. * $P < 0.05$, ** $P < 0.01$, *** $P < 0.001$. NP, n = 35; FGR, n = 35.

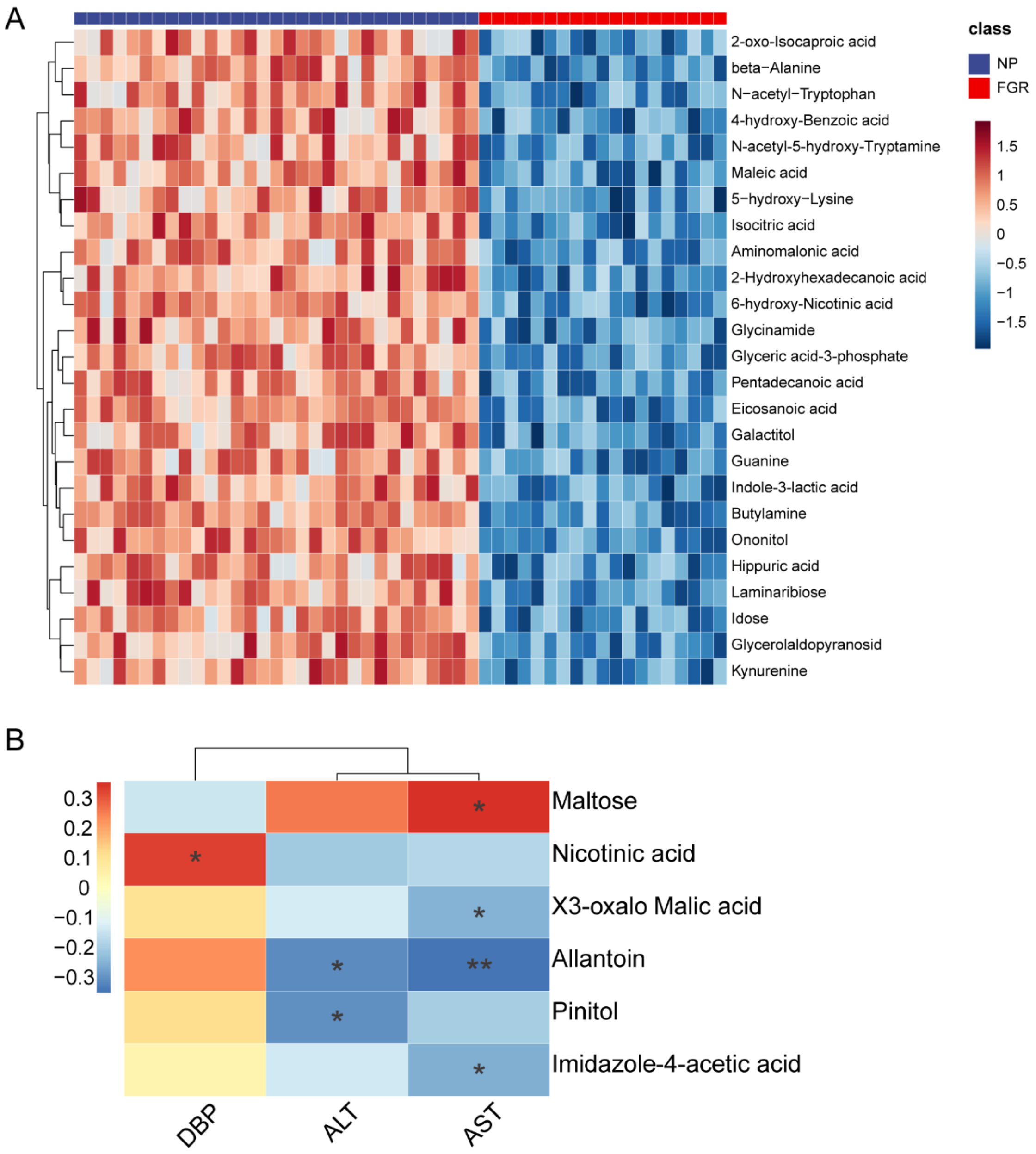

Supplementary Figure 3 (A) The top 25 abundant metabolites. **(B)** Correlation heatmap of maternal serum metabolites and maternal clinical manifestations. Data was processed by Spearman's correlation test. **P* < 0.05, ***P* < 0.01. NP, n = 31; FGR, n = 19.

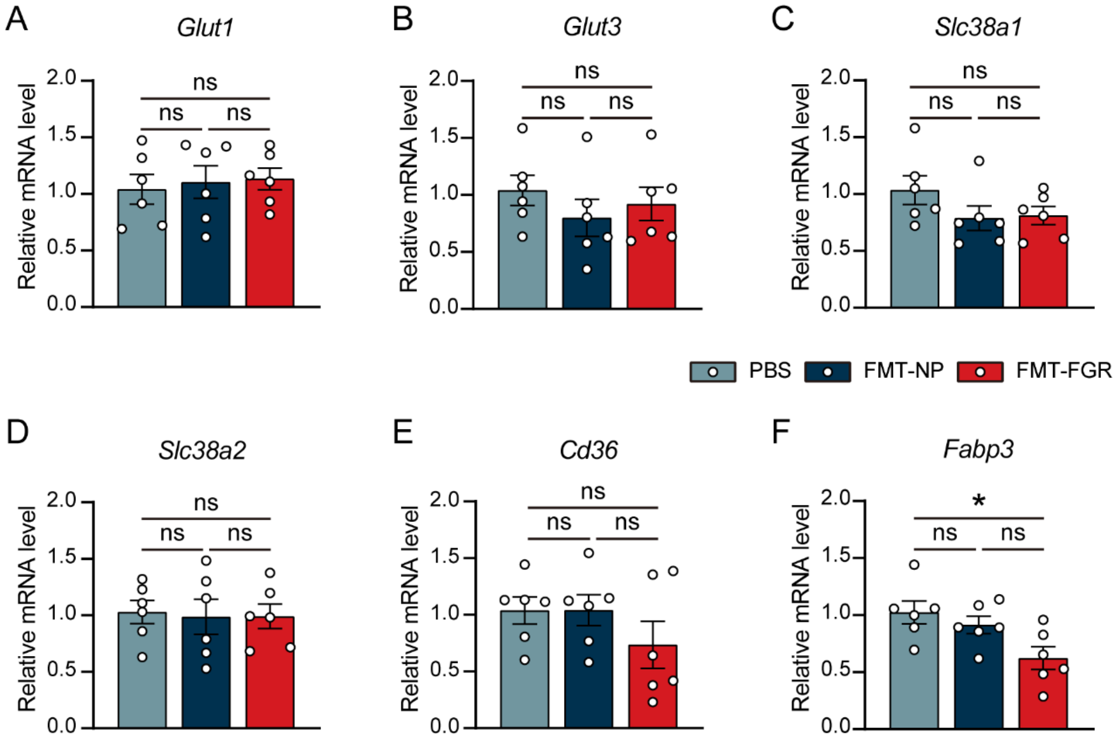

Supplementary Figure 4 The expression of glucose transporters (*Glut1* and *Glut3*), amino acid transporters (*Slc38a1* and *Slc38a2*) and fatty acid transporters (*Cd36* and *Fabp3*) at the mRNA level in the placenta, normalized to *18S*. Data was presented as mean ± SEM. One-way ANOVA test was conducted followed by Bonferroni test. **P* < 0.05, ns, not significant. n = 6 /group.
